# THBS1 identificated as an endometriosis biomarker through evidence from single-cell and bulk transcriptomic profiling

**DOI:** 10.1016/j.isci.2025.114164

**Published:** 2025-11-21

**Authors:** Liqi Zhang, Junyan Ma, Yuhui Sun, Jue Zhu, Huaqing Yan, Yichen Chen, Jing Zhang

**Affiliations:** 1 Department of Gynecology, Ningbo Women & Children’s Hospital, Ningbo, Zhejiang 315010, P.R. China; 2 Women’s Hospital, Zhejiang University School of Medicine, Hangzhou, Zhejiang 310006, P.R. China; 3 Ningbo Medical Center Lihuili Hospital, Ningbo University, Ningbo, Zhejiang 315000, P.R. China

**Keywords:** clinical genetics, female reproductive endocrinology, transcriptomics

## Abstract

Endometriosis affects a substantial number of women of reproductive age, yet current diagnostic methods rely on invasive procedures. To address this limitation, we investigated THBS1 as a potential biomarker and regulator of disease progression. Integrating bulk and single-cell RNA sequencing data, we identified THBS1 as a gene enriched in ectopic endometrial tissues. Immunohistochemistry confirmed its elevated expression, while functional assays revealed that THBS1 promotes endometrial cell proliferation, migration, and invasion. In a subcutaneous xenograft model, THBS1 silencing by small interfering RNA suppressed ectopic lesion growth, demonstrating its functional relevance *in vivo*. These findings position THBS1 as a central modulator of endometrial cellular behavior and provide a conceptual framework linking its molecular activity to the pathophysiology of endometriosis. The study supports THBS1 as a candidate biomarker that could inform the development of noninvasive diagnostic strategies.

## Highlights

• Integrated bulk and single-cell transcriptomics nominate THBS1 in endometriosis
• THBS1 knockdown inhibits cell growth and invasion *in vitro* via TGF-β pathway
• THBS1 knockdown suppresses ectopic endometriosis lesion growth *in vivo*

## Graphical abstract

**Figure:**
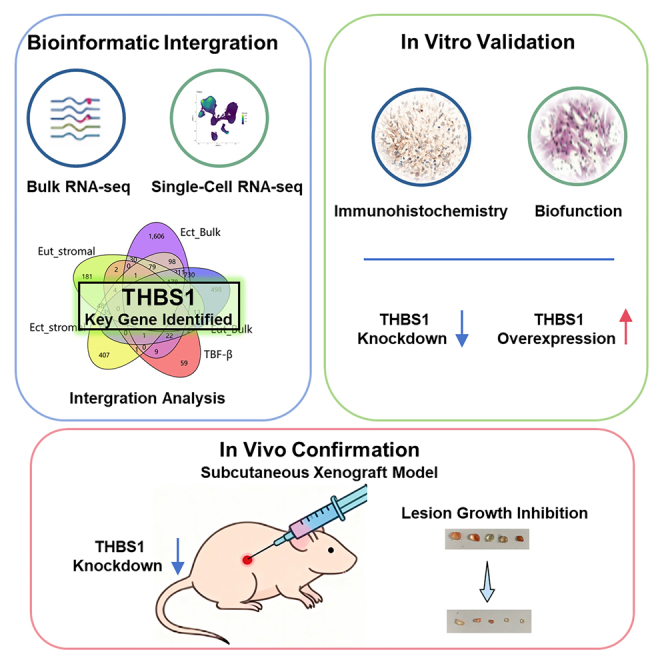

## Introduction

Endometriosis, which affects approximately 10% of women of reproductive age, manifests as dysmenorrhea, non-menstrual pelvic pain, and infertility.^1^^,^^2^ Despite the high global endometriosis prevalence, little is known about the disease, resulting in a diagnostic delay spanning from 4 to 11 years, during which 65% of affected women are initially misdiagnosed due to lack of awareness and non-specific nature of the symptoms.^3^ Moreover, the dependence on surgical diagnosis through laparoscopy presents a substantial barrier to the prompt identification and management of the disease. In addition, laparoscopy is associated with certain diagnostic limitations. Laparoscopy relies on the visual inspection of lesions; however, it cannot detect microscopic vascularization and innervation. Thus, in many instances, disease presence could be missed by laparoscopy.^4^ Furthermore, a normal clinical examination and negative histology do not exclude endometriosis. Occult endometriosis has been documented in incidental peritoneal biopsy specimens, emphasizing the need for diagnostics beyond visible manifestations or histological findings.^5^^,^^6^ Endometriosis can have serious effects on patients’ quality of life and fertility; thus, diagnostic delays hinder early treatment and can be detrimental. Therefore, early identification and management of endometriosis is crucial and can be facilitated by transitioning toward clinical diagnosis as a complement to surgical diagnosis.^7^

The *TGF-β* (transforming growth factor β) pathway is a well-known signaling cascade that plays a crucial role in numerous physiological processes. TGF-β exerts important functions in cell growth,^8^ differentiation,^9^ extracellular matrix production,^10^ and immune regulation.^11^ Under normal physiological conditions, this pathway is activated through the binding of secreted TGF-β to its receptor, which in turn activates downstream signaling molecules such as SMAD proteins to modulate gene expression, thereby maintaining cellular and tissue homeostasis and facilitating tissue repair.^12^ Specifically, the *TGF-β* pathway is believed to contribute pivotally to the development of endometriosis because of its pro-fibrotic role.^13^ TGF-β stimulates the proliferation and differentiation of stromal cells, enhancing their invasive capacity, and may also support the maintenance and growth of ectopic lesions by promoting angiogenesis.^14^ Moreover, the *TGF-β* pathway can modulate the activity of immune cells, potentially creating a local immune environment that is more conducive to the survival of ectopic tissue.^15^ Therefore, targeting the *TGF-β* pathway may offer a therapeutic strategy for the treatment of endometriosis.

Using an integrative bioinformatic analysis approach that combined bulk and single-cell RNA sequencing data, we focused on TGF-β-related genes and differentially expressed genes in ectopic endometrial tissue, eutopic endometrial tissue, and normal endometrial tissue. Through this method, we identified five genes that may play important roles in the progression of endometriosis. Further literature review and integration with our previous single-cell transcriptomic analysis revealed that thrombospondin-1 (THBS1) was significantly upregulated in the ectopic endometrium of patients with endometriosis and may have the potential to serve as a biomarker.^16^ *THBS1*, discovered in 1971, was the first THBS family member to be reported.^17^ THBS1 is synthesized and secreted by various cell types, including endothelial cells, fibroblasts, and smooth muscle cells.^18^^,^^19^^,^^20^ THBS1 encompasses six functional domains: (1) a globular region containing the heparin-binding site at the N-terminus, (2) a segment homologous to type I collagen, (3) three type I repeat sequences resembling properdin, (4) three epidermal growth factor-like type H repeat sequences, (5) seven type III repeat sequences that bind calcium ions, and (6) a cell-binding site located at the C-terminus. Each domain exerts distinct functions by specifically binding to different molecules.^21^ Several factors modulate THBS1 expression. Shi et al.^22^ found that hypoxia inhibits osteogenic differentiation and promotes YTHDF1 expression, which translationally regulates THBS1 in an m6A-dependent manner. Sun et al.^23^ revealed that USF2 knockdown leads to THBS1 downregulation, resulting in the inhibition of the *TGF-β* signaling pathway and a reduction in pyroptosis in sepsis-induced acute kidney injury. THBS1 is also involved in the progression of various human cancers. For example, Xiao et al.^24^ reported that the co-delivery of a THBS1 inhibitor and a checkpoint inhibitor holds promise as a strategy to enhance cancer immunotherapy. Although THBS1 has been extensively studied in tumors, research on its role in endometriosis is limited. Only two investigations referenced THBS1, while neither centered on it as a primary focus.^25^^,^^26^

Therefore, the objective of this study was to further investigate the significance of THBS1 in the endometriosis using *in vitro* and *in vivo* experiments, clinical sample validation, and bioinformatics analysis. We hope that our findings will lay the groundwork for future investigations into potential diagnostic biomarkers and therapeutic targets in endometriosis.

## Results

### Identifying different expressed genes via bulk RNA sequencing data analysis

**Figure 1:**
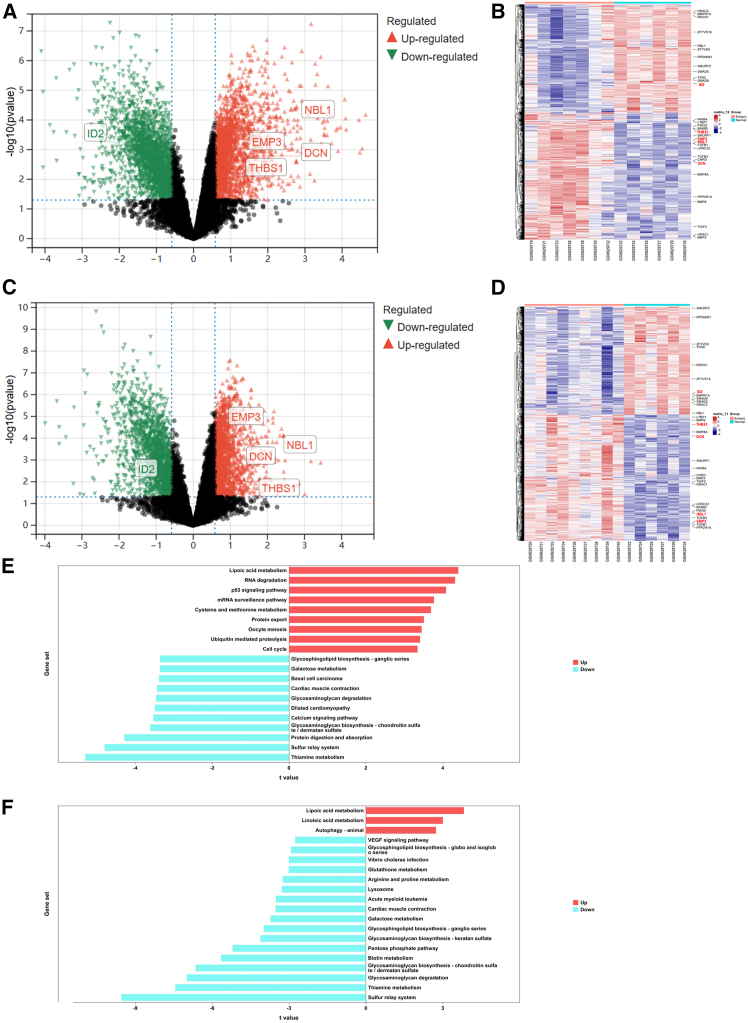
Identifying different expressed genes via bulk RNA sequencing data analysis (A) Upon comparison of ectopic endometrium tissue with normal endometrium, we identified 2,620 upregulated genes and 2,503 downregulated genes. (B) Heatmap showed different expressed genes between ectopic endometrium tissue and normal endometrium. (C) Comparing eutopic endometrium tissue with normal endometrium showed 2,104 upregulated genes and 1,801 downregulated genes. (D) Heatmap showed different expressed genes between eutopic endometrium tissue and normal endometrium. (E) GSVA in comparisons of ectopic endometrium tissue with normal endometrium. (F) GSVA in comparisons of eutopic endometrium tissue with normal endometrium. The absolute magnitude of the *t* statistic reflects the degree of enrichment pathway disparity, with larger values denoting heightened biological significance.

To identify the key genes involved in the pathogenesis of endometriosis, we performed a comprehensive analysis of bulk RNA sequencing data. The GSE25628 dataset was utilized for this purpose, encompassing eight paired samples of eutopic and ectopic endometrium, as well as six samples of normal endometrium. Upon comparison of ectopic endometrium tissue with normal endometrium, we identified 2,620 upregulated genes and 2,503 downregulated genes (Figure 1A). In contrast, when comparing eutopic endometrium tissue with normal endometrium, we detected 2,104 upregulated genes and 1,801 downregulated genes (Figure 1B). Meanwhile, we plotted heatmaps comparing ectopic endometrium tissue with normal endometrium (Figure 1C) and eutopic endometrium tissue with normal endometrium (Figure 1D). We annotated the differentially expressed genes involved in the *TGF-β* signaling pathway to preliminarily explore the key genes associated with the pathogenesis of endometriosis through this pathway.

Next, we performed gene set variation analysis (GSVA) to identify potentially affected biological pathways in comparisons of ectopic endometrium tissue with normal endometrium (Figure 1E) and eutopic endometrium tissue with normal endometrium (Figure 1F). In Figure 1E, ectopic endometrium samples exhibited several significantly upregulated pathways compared with normal endometrium samples, including lipoic acid metabolism and RNA degradation. These upregulated pathways may be associated with enhanced metabolic activity and protein synthesis mechanisms in ectopic endometrium tissue, reflecting its highly active growth characteristics. Downregulated pathways included glycosaminoglycan biosynthesis—chondroitin sulfate, which may indicate changes in matrix construction. In Figure 1F, eutopic endometrium samples also showed upregulation of lipoic acid metabolism and autophagy signaling pathway. These alterations may suggest important regulatory roles of eutopic endometrium in cell survival. Downregulated pathways were related to immune responses, such as bacterial invasion, indicating potential immune suppression in eutopic endometrium. These GSVA results reveal significant differences in biological functions between endometriosis and normal endometrium. The differential expression of these pathways provides important biological clues for selecting research directions and for a deeper understanding of the molecular mechanisms underlying endometriosis.

### Identifying different expressed genes via single-cell RNA sequencing data analysis

**Figure 2:**
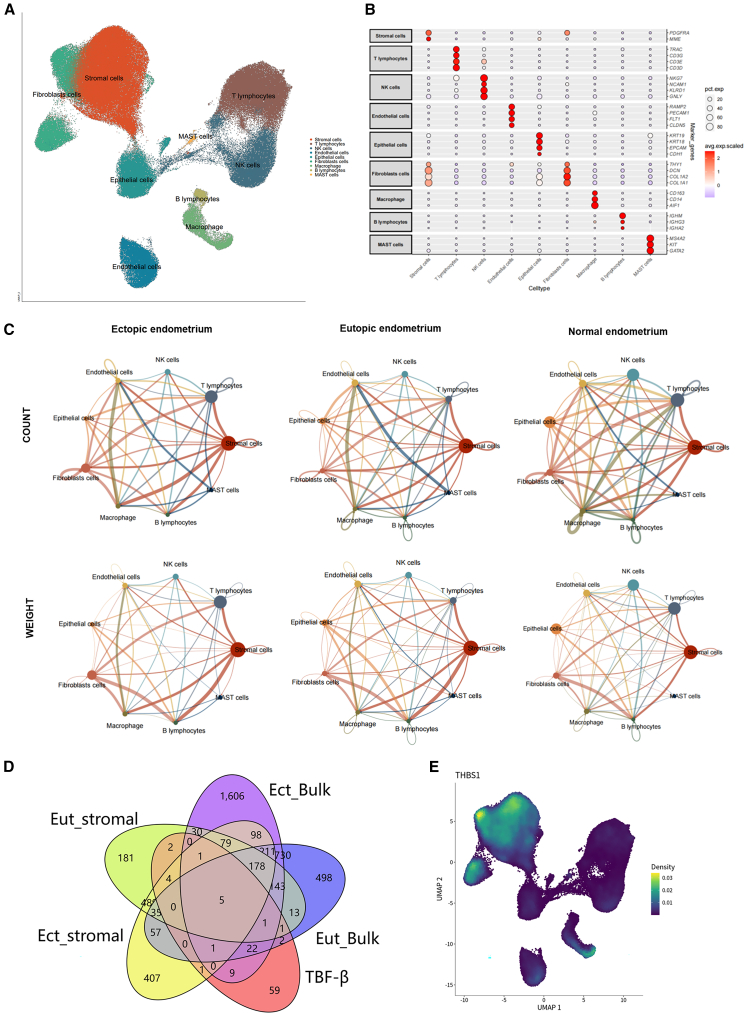
Identifying different expressed genes via single-cell RNA sequencing data analysis (A) UMAP after analyzing single-cell RNA sequencing data from GSE213216, GSE179640, and GSE214411. (B) The marker genes used for annotating the cell clusters. (C) Cell communication analysis among cells in ectopic, eutopic, and normal endometrium. (D) A Venn figure to analysis the key genes in the development of endometriosis using different expressed genes via integrating bulk and single-cell RNA sequencing data analysis. (E) A heatmap illustrating the expression of THBS1 in single-cell RNA sequencing data.

After analyzing single-cell RNA sequencing data from GSE213216, GSE179640, and GSE214411, we identified nine cell types for biological analysis, namely stromal cells, T lymphocytes, NK cells, endothelial cells, epithelial cells, fibroblasts, macrophages, B lymphocytes, and mast cells, as shown in Figure 2A. Stromal cells were found to be the most abundant cell type among all the identified cells. Figure 2B displays the marker genes used for annotating the cell clusters.

Next, we performed cell communication analysis. Through the analysis of cell communication networks, we examined the interactions among cells in ectopic, eutopic, and normal endometrium (Figure 2C). The results showed that stromal cells had significant communication relationships with other cell types, as indicated by both the count and weight analyses, particularly in ectopic and eutopic endometrium. These findings suggest that in endometriosis, stromal cells may contribute to disease pathogenesis by modulating the microenvironment and facilitating cell-cell communication. Therefore, a detailed investigation of stromal cells may provide important insights into the mechanisms underlying endometriosis.

### Identifying key genes in the development of endometriosis

A Venn figure was plotted to analysis the key genes in the development of endometriosis using different expressed genes via integrating bulk and single-cell RNA sequencing data analysis (Figure 2D). Eut_stromal represents the differentially expressed genes between eutopic endometrial stromal cells and normal endometrial stromal cells. Ect_stromal represents the differentially expressed genes between ectopic endometrial stromal cells and normal endometrial stromal cells. Eut_Bulk represents the differentially expressed genes in bulk RNA sequencing data comparing eutopic endometrium and normal endometrium. Ect_Bulk represents the differentially expressed genes in bulk RNA sequencing data comparing ectopic endometrium and normal endometrium. By intersecting the differentially expressed genes from the following comparisons—5,123 genes from Ect_Bulk vs. Normal, 3,905 genes from Eut_Bulk vs. Normal, 1,566 genes from Ect_stromal vs. Normal_stromal, 1,163 genes from Ect_stromal vs. Normal_stromal, and 108 genes in the *TGF-β* signaling pathway—we identified five key genes associated with the *TGF-β* pathway in endometriosis: *NBL1*, *EMP3*, *ID2*, *DCN*, and *THBS1.*

Through further literature review, we found that NBL1 and EMP3 have not been reported in relation to TGF-β. *DCN* has been shown to be associated with endometriosis, but not with TGF-β.^27^ In contrast, ID2 and THBS1 have been documented to directly regulate TGF-β.^28^^,^^29^ However, research on ID2 in endometriosis has already been explored.^28^ Based on these findings, we elected to undertake a comprehensive analysis of THBS1 to elucidate its role in the pathogenesis of endometriosis. Meanwhile, a heatmap was plotted to illustrate the expression of THBS1 in single-cell RNA sequencing data (Figure 2E). Elevated expression of THBS1 occurs primarily within the stromal cells.

### THBS1 expression is elevated in the ectopic endometrial tissues of women with endometriosis

**Figure 3:**
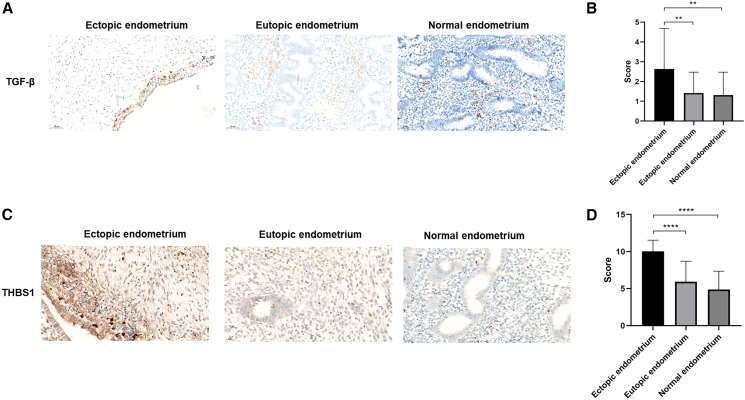
THBS1 expression is elevated in ectopic endometrial tissue (A) Representative immunohistochemical staining for TGF-β. The scale bars located in the lower left corner of the images indicate 50 μm. (B) TGF-β expression was assessed using immunohistochemical staining. (C) Representative immunohistochemical staining for THBS1. The scale bars located in the lower left corner of the images indicate 20 μm. (D) THBS1 expression was assessed using immunohistochemical staining. Error bars represent the SD obtained from three independent experiments. A one-way ANOVA, in conjunction with Dunn’s post hoc test, was used for intergroup comparisons. ∗*p* < 0.05, ∗∗*p* < 0.01, ∗∗∗*p* < 0.001, and ∗∗∗∗*p* < 0.0001.

To determine whether there were any alterations in THBS1 and TGF-β protein expression levels in endometrial tissues, we analyzed eutopic, ectopic, and normal endometrial tissues via immunohistochemical staining analysis. The analysis included 89 participants, comprising 60 patients with endometriosis (unpaired 30 eutopic endometrial samples and 30 ectopic endometrial samples) and 29 controls. Baseline characteristics are detailed in Table 1. Elevated TGF-β and THBS1 expression was observed in the ectopic endometrium of patients with endometriosis (Figures 3A–3D). As shown in Figures 3A and 3B, immunohistochemical staining and corresponding score analysis revealed that the expression levels of TGF-β in ectopic endometrium were significantly higher than those in either eutopic endometrium or normal endometrium. Similarly, as depicted in Figures 3C and 3D, the expression levels of THBS1 mirrored those of TGF-β, with significantly elevated levels in ectopic endometrium compared with eutopic and normal endometrium. Additionally, western blot analysis was performed in cells from ectopic endometrium tissues and demonstrated a marked reduction in TGF-β1 expression following si-THBS1 transfection, indicating that suppression of THBS1 attenuates activation of the TGF-β signaling pathway (Figure S1).

**Table 1:** Baseline clinical characteristics of patients included in immunohistochemical analysis

Clinical characteristic	Control endometrium	Eutopic endometrium	Ectopic endometrium	*p* value
Patients	29	30	30	–
Age (years)	30.90 ± 4.79	31.63 ± 4.06	32.90 ± 4.75	0.24
BMI (kg/m^2^)	21.19 ± 2.72	21.26 ± 4.66	21.78 ± 3.54	0.80
Endometrial thickness (cm)	0.62 ± 0.27	0.66 ± 0.32	0.65 ± 0.32	0.87

### THBS1 modulates *in vitro* proliferation, migration, and invasion

**Figure 4:**
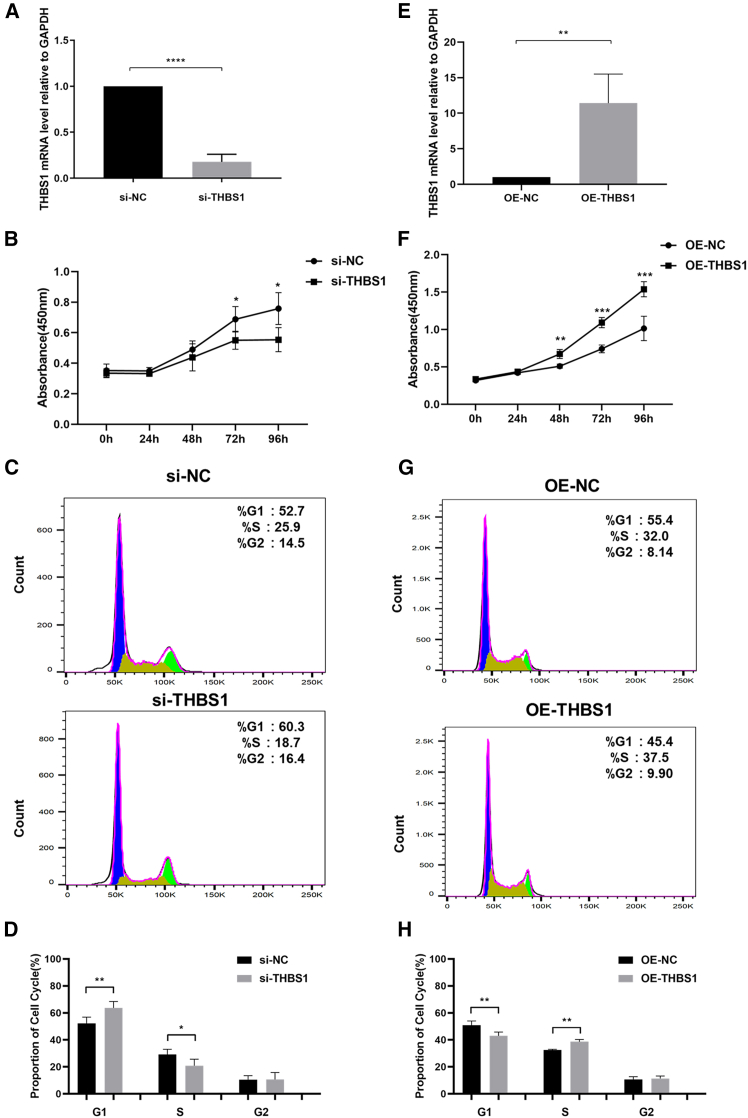
THBS1 modulates the proliferation of cells *in vitro* (A) qPCR assays were performed to confirm the siRNA transfection effect. (B) Comparison of five-day cell proliferation between the si-NC and si-THBS1 groups via Cell Counting Kit-8 assay. (C) Representative images of cell cycle analysis between the si-NC and si-THBS1 groups. (D) Comparison of the proportion of cells at different phases of the cell cycle between the si-NC and si-THBS1 groups. (E) qPCR assay was performed to confirm the lentivirus transfection effect. (F) Comparison of five-day cell proliferation between the overexpression negative control (OE-NC) and OE-THBS1 groups via Cell Counting Kit-8 assay. (G) Representative images of cell cycle analysis between the OE-NC and OE-THBS1 groups. (H) Comparison of the proportion of cells at different phases of the cell cycle between the OE-NC and OE-THBS1 groups. Error bars represent the SD obtained from three independent experiments. Comparisons between two groups were performed using the Student’s *t* test. ∗*p* < 0.05, ∗∗*p* < 0.01, ∗∗∗*p* < 0.001, ∗∗∗∗*p* < 0.0001.

To investigate the impact of THBS1 on the progression of endometriosis, the effects of THBS1 were examined in cells from ectopic and eutopic endometrium tissues. Because THBS1 expression was significantly higher in ectopic tissues than in eutopic tissues, we conducted knockdown experiments in cells from ectopic tissues and overexpression experiments in eutopic tissues. First, we conducted qPCR to confirm that small interfering RNA (siRNA) could inhibit THBS1 expression at the RNA level in cells from ectopic tissues (Figure 4A). Similarly, the lentivirus-mediated overexpression of THBS1 was confirmed in cells from eutopic tissues (Figure 4E). The proliferative capacity of cells in the si-THBS1 group was lower than that in the si-NC group (Figure 4B). In addition, flow cytometry was performed to assess changes in the cell cycle at 72 h post-transfection. THBS1 knockdown resulted in cell-cycle arrest at the G1 phase (Figures 4C and 4D). Conversely, THBS1 overexpression enhanced cell viability, reduced the proportion of cells in G1 phase, and increased the proportion of cells in S phase (Figures 4F, 4G, and 4H).

**Figure 5:**
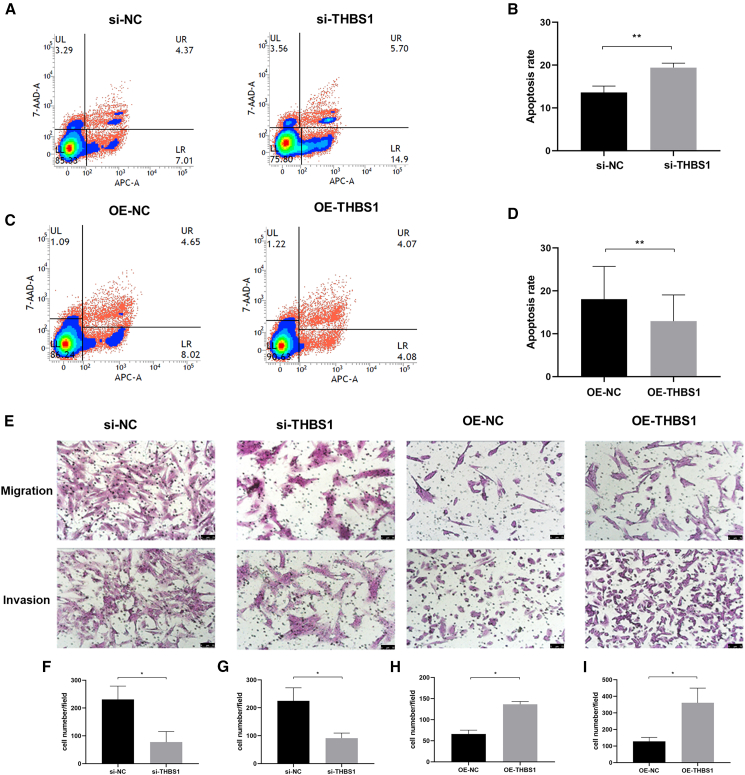
THBS1 modulates *in vitro* apoptosis, migration, and invasion (A) Representative images of flow cytometry showing an increased apoptosis rate in cells following THBS1 knockdown. (B) THBS1 knockdown increased the apoptosis rate. (C) Representative images of flow cytometry showing a reduced apoptosis rate in cells following THBS1 overexpression. (D) THBS1 overexpression reduced the apoptosis rate. (E) Representative images of the transwell assay. The scale bars located in the lower right corner of the images indicate 75 μm. (F) THBS1 knockdown inhibited the migration capacity. (G) THBS1 knockdown inhibited the invasive capacity. (H) THBS1 overexpression promoted the migration capacity. (I) THBS1 overexpression promoted the invasive capacity. Error bars represent the SD obtained from three independent experiments. Comparisons between two groups were performed using the Student’s *t* test. ∗*p* < 0.05, ∗∗*p* < 0.01.

Next, we investigated the effect of THBS1 on the apoptosis of cells using flow cytometry. Consistent with the results of the aforementioned approach, THBS1 was knocked down in cells from ectopic tissues and overexpressed in cells from eutopic tissues. THBS1 knockdown increased cell apoptosis, with a particularly notable change in early apoptosis (Figures 5A and 5B). Conversely, THBS1 overexpression reduced the cell apoptotic rate, primarily during early apoptosis (Figures 5C and 5D). These findings indicate that elevated THBS1 expression inhibits apoptosis.

Moreover, we assessed the impact of THBS1 on cell migration and invasion capacity using transwell assays. THBS1 knockdown resulted in a significant reduction in the number of cells in migration and invasion assay (Figures 5E, 5F, and 5G). Conversely, THBS1 overexpression increased cell migration and invasion (Figures 5E, 5H, and 5I). These findings indicate that THBS1 modulates the migration and invasive capacity of cells.

### THBS1 knockdown significantly inhibited development of ectopic endometriosis *in vivo*

**Figure 6:**
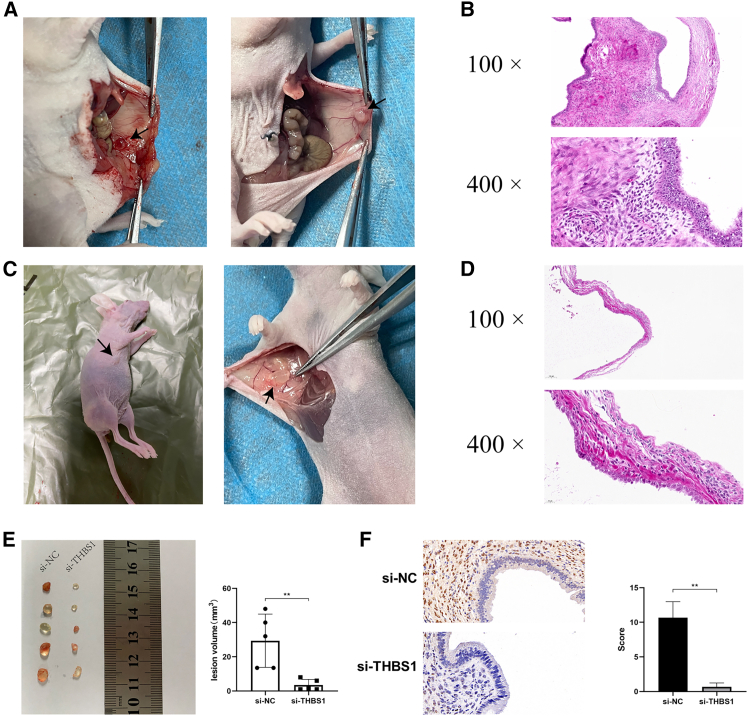
THBS1 knockdown significantly inhibited the proliferation of ectopic endometriosis *in vivo* (A) Successful ectopic lesions were observed in the intraperitoneal xenograft model in nude mice. (B) Representative image from the pathological examination of the ectopic lesions in the intraperitoneal xenograft model. (C) Successful ectopic lesions observed in the subcutaneous xenograft model in nude mice. (D) Representative image from the pathological examination of the ectopic lesions in the subcutaneous xenograft model. (E) THBS1 siRNA inhibited the growth of ectopic lesions. (F) Immunohistochemical staining confirmed that the si-THBS1 group exhibited significantly reduced THBS1 expression. The scale bars located in the lower left corner of the images indicate 20 μm. Error bars represent the SD obtained from three independent experiments. Comparisons between two groups were performed using the Student’s *t* test. ∗∗*p* < 0.01.

To investigate the effects of THBS1 *in vivo*, we established an endometriosis mouse model. We established two different models: an intraperitoneal xenograft model and a subcutaneous xenograft model, to investigate the formation of ectopic lesions in endometriosis. The successfully modeled ectopic lesions in the intraperitoneal xenograft model in nude mice appeared as macroscopically visible vesicles containing clear fluid or yellow-brown turbid fluid, accompanied by surrounding angiogenesis (Figure 6A). Pathological examination revealed that the lesions consisted of glandular epithelial cells and stromal fibroblasts (Figure 6B). Out of the 10 nude mice, a total of 10 transplanted lesions were generated, with successful xenograft achieved in four instances, resulting in a success rate of 40%. The ectopic lesions in the subcutaneous xenograft model in nude mice appeared as subcutaneous cystic elevations with a soft texture. Upon dissection, macroscopic examination revealed vesicular lesions containing clear or yellow-brown fluid, accompanied by surrounding blood vessels (Figure 6C). Histopathological analysis revealed glandular epithelial cells and stromal fibroblasts within the lesions (Figure 6D). Ten transplanted lesions were generated in 10 nude mice, with eight instances showing successful modeling. In the remaining two cases, no apparent residual lesions were observed subcutaneously, resulting in an overall success rate of 80%.

The intraperitoneal xenograft model more closely mimics the peritoneal environment of endometriosis but was limited by a success rate of approximately 40%, which reduced its reproducibility. By contrast, the subcutaneous model achieved an 80% success rate and allowed more consistent evaluation of lesion growth and cellular behavior, thereby providing a stable platform for *in vivo* validation. We recognize that this approach sacrifices some anatomic fidelity, and that alternative systems such as patient-derived organoids may offer complementary insights. Nonetheless, for the purposes of this study, the subcutaneous model represented the most feasible option for functional assessment of THBS1.

Sixteen nude mice were selected and fragments of human endometrium were ectopically implanted in the axillary region with intramuscular injection of estrogen for maintenance. After two weeks of modeling, surface observation revealed successful modeling in 10 nude mice, which were then randomly divided into si-NC and si-THBS1 groups, with five mice in each group. Subsequently, si-NC and si-THBS1 were administered to the respective groups. After eight administrations, the mice were euthanized, and macroscopic examination confirmed successful modeling in all 10 mice. Upon isolation of the lesions, it was observed that THBS1 siRNA inhibited the growth of ectopic lesions, as the lesion volume in the si-THBS1 group was significantly lower than that in the si-NC group (Figure 6E). Immunohistochemical staining demonstrated positive THBS1 expression in all lesions, whereas the si-THBS1 group exhibited significantly reduced THBS1 expression (Figure 6F).

## Discussion

Endometriosis is a primary cause of infertility and pelvic pain in women. The classic theory of retrograde menstruation was initially proposed in 1921 and has gained widespread recognition.^30^ However, the majority of women experience some degree of retrograde menstruation, while only 10% develop endometriosis.^31^ Increasing evidence suggests that ectopic endometrial lesions undergo several molecular alterations. Significant differences in mRNA and non-coding RNA profiles are found in the ectopic endometrium and are associated with distinct biological behaviors such as cell adhesion and invasion.^32^^,^^33^^,^^34^ These gene expression differences may contribute to the development of ectopic lesions.^35^ Analyzing the biological functions of endometrial stromal cells and identifying important genes that may be involved in these functions can provide insight into the pathogenesis and future treatment of endometriosis.

*THBS1* is a multifunctional gene that engages in a wide range of cellular interactions with the surrounding extracellular matrix via receptor binding or modulation of extracellular proteinases.^36^ THBS1 has been extensively documented in various human diseases and tumors.^37^ There intricate mechanisms of THBS1 in various diseases remain controversial. Previously, we identified THBS1 in endometrial stromal cells.^16^ THBS1 repeats exert anti-angiogenic effects.^38^ In addition, THBS1 affects wound healing.^39^ Furthermore, studies suggest that THBS1 supports the adhesion and migration of tumor cells *in vitro*.^40^ In gynecological tumors, plasma levels of THBS1 are associated with tumor grading.^41^ However, the mechanisms underlying its role in endometriosis remain poorly understood. Therefore, we aimed to elucidate the relationship between THBS1 and endometriosis. In this study, we evaluated the functional role of THBS1 in modulating endometrial proliferation, migration, and invasion.

We based our bulk RNA-seq analysis on the GSE25628 dataset, which included paired eutopic and ectopic samples together with normal controls. This choice ensured internal consistency but limited the number of datasets available for analysis. Reliance on a single dataset may constrain generalizability, and findings must therefore be interpreted with caution. Integrating multiple cohorts often introduces substantial batch effects, reflecting differences in sample processing, sequencing platforms, and analytic pipelines. Such heterogeneity, if not rigorously corrected, can obscure true biological variation. By focusing on a uniform dataset, we sought to reduce technical noise and highlight biologically meaningful differences. Nonetheless, the possibility of dataset-specific bias remains, and future studies using integrated multi-cohort analyses with robust correction methods, complemented by single-cell data, will be important to confirm and extend our findings.

There has been extensive research into the *TGF-β* signaling pathway, as it plays a crucial role in processes including tumor development, embryonic development, organ formation, wound healing, extracellular matrix production, and immune function.^42^ A significant increase in TGF-β1 levels and activation of the *TGF-β* signaling pathway have been observed in endometriosis; these changes in turn promote the survival, adhesion, proliferation, and invasiveness of ectopic endometrial tissue.^43^^,^^44^ In endometriosis, the *TGF-β* signaling pathway can activate the AKT signaling pathway by promoting AKT phosphorylation.^45^ It can also activate the Wnt/β-Catenin pathway in a paracrine manner, facilitating fibrosis and mediating the EMT.^46^^,^^47^ In mouse models of endometriosis, TGF-β mediates disease progression via the SMAD signaling pathway, and inhibiting TGF-β can effectively alleviate disease progression.^48^ So, our bioinformatic analysis selected TGF-β related genes as a target.

Next, we identified five key genes associated with the *TGF-β* pathway in endometriosis: *NBL1*, *EMP3*, *ID2*, *DCN*, and *THBS1.* Although all these genes may be identified as potential biomarkers in endometriosis progression, we prioritized THBS1 for further analysis due to prior evidence and its comparative status among key biomarkers. Through lab verification, we revealed the potential role of THBS1 in contributing to the progress of endometriosis. Overall, our study reveals a significant increase in THBS1 level in ectopic endometrium tissues and validates the potential of THBS1 as a biomarker in endometriosis. These findings provide valuable support for understanding the occurrence and progression of endometriosis and hold promise for future therapeutic interventions.

Our analysis emphasized stromal cells, in which THBS1 showed the most consistent and biologically relevant changes. Other cell types within the endometriotic microenvironment, including macrophages and endothelial cells, may also contribute to disease progression through mechanisms such as immune modulation and angiogenesis. A deeper exploration of these populations was beyond the scope of this study, but such analyses will be important in future work to clarify the broader cellular context of THBS1 activity and to identify additional pathways that may drive endometriosis.

Overall, our findings elucidate the role of THBS1 in endometriosis and reveal, both *in vivo* and *in vitro*, that THBS1 affects the proliferation, migration, and invasion of cells. THBS1 may serve as a potential diagnostic biomarker for endometriosis. Nonetheless, further investigations are warranted to unravel the precise mechanism by which THBS1 contributes to the progression of endometriosis.

### Limitations of the study

Our study has some limitations. First, our identification of THBS1 was based on bioinformatics analysis of existing data combined with a literature review. This approach inevitably introduces selection bias, and we cannot assert that THBS1 is the most critical factor in the pathogenesis of endometriosis. Second, our analysis was based on a single bulk RNA-seq dataset (GSE25628), which ensured internal consistency but may limit generalizability. Integration of multiple cohorts could introduce batch effects and platform-related biases, yet future studies with robust correction methods and complementary single-cell data will be needed to validate and extend our findings. Third, although we successfully established a subcutaneous xenograft model in nude mice, the alterations in the immune environment of the immunodeficient mice were not suitable for immunological investigations. Finally, in the *in vivo* experiments, due to the small size of the endometriosis lesions, significant errors were encountered during the continuous measurement process, preventing the establishment of a growth curve. Although the intraperitoneal endometriosis model enables better simulation of the peritoneal growth environment of patients with endometriosis, the laparotomy procedure causes significant damage to nude mice. Additionally, the success of the model can be determined only after euthanizing the nude mice and performing a laparotomy. Therefore, a subcutaneous modeling approach was selected for our study.

## Resource availability

### Lead contact

Requests for further information and resources should be directed to and will be fulfilled by the lead contact, Jing Zhang, zhangjyy1978@163.com.

### Materials availability

Materials generated in this study will be made available from the lead contact upon reasonable request and in accordance with institutional and ethical guidelines of The Women’s Hospital affiliated with Zhejiang University.

### Data and code availability

• Data reported in this paper will be shared by the lead contact upon request.
• This paper is based on previously published and deposited sequencing data and does not report original code. The relevant accession numbers are included in the key resources table.
• Any additional information required to reanalyze the data reported in this paper is available from the lead contact upon reasonable request.

## Acknowledgments

This study was supported by Natural Science Foundation of Ningbo (grant no. 2023J145); Ningbo Clinical Medical Research Center for Gynecological Diseases (grant no. 2024L002); and Ningbo Top Medical and Health Research Program (no. 2024021020). We would like to thank Editage (www.editage.cn) for English language editing and the CNSKnowall platform (https://cnsknowall.com) for supporting with bioinformatic analysis.

## Author contributions

H.Y. and L.Z. designed this study; L.Z. drafted the manuscript; J.M., Y.S., and J.Z. collected the clinical data; J.Z. provided critical comments and suggestions and revised the manuscript.

## Declaration of interests

The authors declare no competing interests.

## STAR★Methods

### Key resources table

**Table:** 

REAGENT or RESOURCE	SOURCE	IDENTIFIER
**Antibodies**		

THBS1	Cell Signaling Technology	Cat# 37879, RRID:AB_2799123
TGF-β	Affinity Biosciences	Cat# BF8012, RRID:AB_3665500
β-actin	Affinity Biosciences	Cat# AF7018, RRID:AB_2839420

**Bacterial and virus strains**		

pSLentiSFH-EGFP-P2A-Puro-CMV-THBS1-3xFLAG-WPRE	OBiO Technology	N/A

**Biological samples**		

Endometrial tissue samples	The Women’s Hospital Affiliated with Zhejiang University	N/A

**Chemicals, peptides, and recombinant proteins**		

DMEM/F-12	Gibco, USA	Cat#12634010
Fetal Bovine Serum	Gibco, USA	Cat#16140071

**Critical commercial assays**		

BCA kit	Beyotime	Cat#P0010S
Cell Cycle Staining Kit	MultiSciences	Cat#CCS012
Annexin V-APC/7-AAD Apoptosis Kit	MultiSciences	Cat#AP105

**Deposited data**		

Bulk RNA-seq data	Crispi et al.^49^	GSE25628
Single-cell RNA-seq data	Fonseca et al.^50^	GSE213216
Single-cell RNA-seq data	Tan et al.^51^	GSE179640
Single-cell RNA-seq data	Huang et al.^52^	GSE214411

**Experimental models: Organisms/strains**		

Female BALB/c nude mice	Shanghai SLAC Laboratory Animal	N/A

**Oligonucleotides**		

GAPDH Forward 5′-GGAGCGAGATCCCTCCAAAAT-3′	This paper	N/A
GAPDH Reverse 5′-GGCTGTTGTCATACTTCTCATGG-3′	This paper	N/A
THBS1 Forward 5′-GCCATCCGCACTAACTACATT-3′	This paper	N/A
THBS1 Reverse 5′-TCCGTTGTGATAGCATAGGGG-3′	This paper	N/A
SiRNA targeting sequence for THBS1 5′-GGCUGACCAUGACAAAGAUTT-3	This paper	N/A

**Recombinant DNA**		

H19175 (pSLentiSFH-EGFP-P2A-Puro-CMV-THBS1-3xFLAG-WPRE)	OBiO Technology	N/A

**Software and algorithms**		

R programming version 4.3	The R Project for Statistical Computing	https://www.r-project.org/
GraphPad Prism 8	GraphPad Software	https://www.graphpad.com/
SPSS 17.0	IBM Corp.	https://www.ibm.com/cn-zh/spss

### Experimental model and study participant details

#### Animals

Female BALB/c nude mice aged 6–8 weeks and weighing 16–18 g were obtained from Shanghai SLAC Laboratory Animal Co., Ltd. (Shanghai, China). The mice were sexually mature, had not mated before and were healthy. The mice were housed in a specific pathogen-free (SPF) environment. They were allowed to acclimate for one week. Subsequently, a muscle injection of 0.5 μg of estrogen was administered to each mouse every three days.

After three injections, an intraperitoneal endometriosis xenograft model was established by implanting human eutopic endometrial fragments obtained from women with endometriosis into the peritoneal cavity of the mice. Estrogen muscle injections were administered to maintain the lesions. After four weeks, the mice were euthanized, and the lesions were observed via laparotomy. For the subcutaneous endometriosis lesion model, eutopic endometrial fragments obtained from women with endometriosis were implanted into the axillary region of nude mice, and estrogen muscle injections were administered to sustain the lesions. The mice were randomly divided into si-NC and si-THBS1 groups. Following a two-week modeling period, a total of 1 OD of small interfering RNA (si-NC or si-THBS1) was dissolved in 60 μL of physiological saline and administered via subcutaneous local injection to the two groups of nude mice. The injections were administered every two days until eight administrations were performed. The development of subcutaneous nodules in the mice was monitored. After 33 days of modeling, the nude mice were euthanized, and the axillary lesions were dissected. Endometriotic lesions were subjected to hematoxylin and IHC staining. All animal procedures were conducted in accordance with institutional guidelines and approved by the Women’s Hospital, Zhejiang University School of Medicine (Approval Number: IACUC-20211018-08).

#### Human subjects

Ectopic and eutopic endometrial tissue samples were collected from patients diagnosed with endometriosis, along with endometrial tissue samples from patients without endometriosis who were admitted to the Gynecology Department of The Women’s Hospital Affiliated to Zhejiang University (Hangzhou, China) between 2017 and 2021. Tissue samples were collected during the proliferative phase of the menstrual cycle, as determined by patient history. Endometriosis diagnosis was made using laparoscopic examination and histopathological analysis. The control group consisted of patients with either a uterine septum or primary infertility admitted without a pre-existing diagnosis of endometriosis or adenomyosis. All included patients had regular menstrual cycles, had not received any hormone treatment in the three months before surgery, and had no history of pregnancy within the past six months. They were excluded if they had uterine adenomyosis, severe internal or surgical diseases, autoimmune diseases, severe infections, malignant tumors, or other hormone-dependent diseases. The excised clinical specimens, obtained via laparoscopic and hysteroscopic surgeries, were immediately placed in transport medium and stored at 4°C. Endometrial stromal cells were isolated and cultured within 1 h to initiate primary cell cultures. Alternatively, within half an hour, samples were transferred and stored at −80°C until further use. The experimental procedure followed aseptic principles. Some of the specimens were preserved in polyformaldehyde for subsequent paraffin embedding.

All experiments concerning tissue samples and human blood were approved by the Ethics Committee of The Women’s Hospital Affiliated with Zhejiang University (approval number: 20190037). Prior to sample collection, all the participants provided written informed consent.

### Method details

#### Bulk RNA sequencing data analysis

For the bulk RNA analysis in this study, datasets were retrieved from the Gene Expression Omnibus (GEO) database (https://www.ncbi.nlm.nih.gov/geo). Specifically, we only included the bulk RNA datasets with paired samples of eutopic and ectopic endometrium from the same patients with endometriosis, as well as normal endometrial samples from healthy controls, for study rigor. Initially, four datasets were downloaded, including GSE118928,^53^ GSE86534,^54^ GSE25628,^49^ and GSE7305.^55^ The GSE118928 dataset was excluded due to unmatched samples (a ratio of 7:4 for ectopic to eutopic endometrium). The GSE86534 dataset was excluded because it did not include endometrial samples from healthy controls. The GSE7305 dataset was excluded as it lacked eutopic endometrial samples. Consequently, the GSE25628 dataset was used for subsequent analyses, including 8 paired samples of eutopic and ectopic endometrium, and 6 samples of normal endometrium. Differential expression analyses were performed comparing ectopic endometrium versus normal endometrium and eutopic endometrium versus normal endometrium (ectopic vs. normal, eutopic vs. normal) via Limma package (version 3.40.2) in R 4.3. Differentially expressed genes were identified using a threshold of |logFC| > 0.5 and adjusted *p* value <0.05. GSVA (gene set variation analysis) was performed comparing ectopic endometrium with normal endometrium and eutopic endometrium with normal endometrium.^56^ The GSVA package (v1.34.0) was used to convert the gene expression matrix into a pathway matrix. The differentially enriched pathways were identified from those cataloged in the KEGG (Kyoto Encyclopedia of Genes and Genomes) database (https://www.kegg.jp/kegg/brite.html).^57^

To further investigate TGF-β-related differentially expressed genes, we utilized the KEGGREST function of the R software to retrieve TGF-β-related genes from the human gene database of the KEGG database. A total of 108 TGF-β related genes were identified and downloaded using the pathway identifier hsa04350.

#### Single-cell RNA sequencing data analysis

Single-cell transcriptomic datasets for endometriosis were retrieved from the GEO database, comprising three datasets: GSE213216,^50^ GSE179640,^51^ and GSE214411.^52^ These datasets encompassed three groups: ectopic endometrium from patients with endometriosis, eutopic endometrium from patients with endometriosis, and normal endometrium from healthy individuals. In total, 45 single-cell transcriptomic samples were included. Single-cell RNA sequencing datasets were directly downloaded from the GEO database. The Seurat v4 package was used to read the data.^58^

Cells from the merged samples were filtered based on the following criteria: 300 < nFeature_RNA <2000, nCount_RNA <40,000, percent.mt (percentage of mitochondrial genes) < 30, and log10GenesPerUMI >0.85. The Seurat pipeline was then applied to the combined cell data for dimensionality reduction. Principal component analysis (PCA) was initially used to visualize the main components. The Harmony algorithm was employed to correct for batch effects across different samples. Subsequently, dimensionality reduction was performed using the UMAP and t-SNE methods based on the selected number of principal components. The dataset was annotated with cell types, including T lymphocytes, B lymphocytes, mast cells, fibroblasts, stromal cells, epithelial cells, and endothelial cells, based on a combination of information from the CellMarker 2.0 database and literature review. Marker gene bubble plots and UMAP annotation plots were generated to visualize the distribution and characteristics of these cell types.

Differential expression analysis was performed using the FindAllMarkers function, focusing on identifying differentially expressed genes within and between clusters across different groups. The threshold for identifying differentially expressed genes was set at |logFC| > 0.25 and adjusted *p* value <0.05. Cell communication analysis was conducted using the CellChat package, with the samples divided into three groups: eutopic endometrium, ectopic endometrium, and normal endometrium. This analysis aimed to elucidate the communication networks within each group.

#### Venn diagram depicting

Venn diagram were constructed using the CNSKnowall platform (https://cnsknowall.com). We performed intersection analyses across five distinct gene sets: (1) differentially expressed genes (DEGs) identified in eutopic endometrium compared with normal endometrium through bulk RNA sequencing; (2) DEGs distinguishing eutopic and normal endometrium via single-cell RNA sequencing; (3) DEGs characterizing ectopic endometrium versus normal endometrium using bulk RNA sequencing; (4) DEGs differentiating ectopic and normal endometrium based on single-cell RNA sequencing; and (5) genes functionally related to the *TGF-β* signaling pathway.

#### Cell culture

Endometrial tissues, obtained from women who underwent laparoscopic surgery, were placed in DMEM/F-12 (Gibco, USA). Next, the tissues were finely shredded and sufficiently digested with collagenase I (Life Technologies, USA) in a 37°C water bath for 1 h. Subsequently, the residual tissue fragments and impurities were separated using a 70 μm filtered mesh. The cells were collected by centrifugation. The cell pellet was resuspended and then transferred to six-well plates and incubated at 37°C and 5% CO_2_.

#### cDNA synthesis and real-time quantitative PCR

The cDNA synthesis and real-time quantitative PCR were performed as previously described.^59^ The gene GAPDH was chosen as the reference gene for standardization, and the primer sequences used for the qPCR were obtained using PrimerBank. The primer sequences were synthesized by Generay (Shanghai, China) as follows: GAPDH Forward, 5′-GGAGCGAGATCCCTCCAAAAT-3′; GAPDH Reverse, 5′-GGCTGTTGTCATACTTCTCATGG-3′; THBS1 Forward, 5′-GCCATCCGCACTAACTACATT-3′; THBS1 Reverse, 5′-TCCGTTGTGATAGCATAGGGG-3′. The 2^−ΔΔCt^ method was employed to determine the relative mRNA expression levels of the target genes. All experiments were performed in triplicate.

#### Western blot analysis

Proteins analyzed in this investigation were isolated from transfected cell lines using radioimmunoprecipitation assay (RIPA) lysis buffer. Protein concentrations were determined by means of a bicinchoninic acid (BCA) assay kit. The samples were subsequently resolved on 10% sodium dodecyl sulfate–polyacrylamide gels and subjected to electrophoresis until complete separation was achieved. The proteins were then transferred onto polyvinylidene difluoride (PVDF) membranes by a wet transfer procedure. After blocking with 5% nonfat milk for 1 h, the membranes were incubated overnight at 4°C with primary antibodies (diluted 1:1000). Following three washes with Tris-buffered saline containing Tween 20 (TBS-T) for a cumulative duration of 30 min, the membranes were incubated with horseradish peroxidase–conjugated secondary antibodies (diluted 1:5000) for 1 h at ambient temperature. After additional washes in TBS-T, immunoreactive bands were visualized using an enhanced chemiluminescence detection system (Pierce Biotechnology, Rockford, IL, USA). The primary antibodies used included rabbit anti-THBS1 (Cell Signaling Technology, product number #37879), anti-TGF-β (Affinity Biosciences, product number #BF8012), anti-β-actin (Affinity Biosciences, product number #AF7018).

#### Immunohistochemistry staining

We performed immunohistochemistry staining as previously described.^59^ The antibodies utilized were rabbit antibody against THBS1 (dilution 1:100, ab1823; Abcam) and rabbit antibody against TGF-β1 (dilution 1:100, RAB0238; Maxim Biotech, China). Briefly, we implemented a standardized approach for processing the original data, as follows.

(1) The staining intensity index was categorized into four levels: 0 points (negative), 1 point (1+), 2 points (2+), and 3 points (3+).
(2) The positive rate of staining was classified into five levels: 0 points (negative), 1 point (1–25%), 2 points (26–50%), 3 points (51–75%), and 4 points (76–100%).
(3) The total score was calculated by multiplying the scores of staining intensity and positive rate.

#### Small interfering RNA, lentiviral synthesis, and transfection

THBS1 small interfering RNA (si-THBS1) and negative control siRNA (si-NC) were synthesized by GenePharma Corporation (China). The specific siRNA sequences used were as follows: si-THBS1-sense, 5′-GGCUGACCAUGACAAAGAUTT-3; si-THBS1-antisense, 5′- AUCUUUGUCAUGGUCAGCCTT-3′; si-Negative Control-sense, 5′-UUCUCCGAACGUGUCACGUTT-3′; si-Negative Control-antisense, 5′-ACGUGACACGUUCGGAGAATT-3′. Cells at an approximate confluency of 70%, were placed into six-well plates. The siRNA transfection process was performed using RNA-iMAX (Life Technologies) following the manufacturer’s instructions. At 72 h post-transfection, total protein was carefully extracted, and transfection efficiency was determined using western blotting, as previously described.^60^

THBS1 overexpressing lentivirus (OE-THBS1) and negative control lentivirus (OE-NC) were synthesized by OBiO Technology (China). The lentiviral vector used in this study was H19175 (pSLentiSFH-EGFP-P2A-Puro-CMV-THBS1-3xFLAG-WPRE). Cells were seeded in a 6-well plate, observed daily until approximately 70% confluency was achieved, then transfected. The transfection reagent was prepared in a sterile 1.5 mL centrifuge tube as follows: DMEM/F12 (1 mL), overexpression lentivirus (multiplicity of infection (MOI) × number of cells at the time of infection/viral titer × 1000), and polybrene (5 μg/mL) and mixed thoroughly. The cells in the 6-well plate were aspirated from the original culture medium, and 1 mL of the transfection mixture was added to each well (at an MOI of 30). After 24 h, the medium was replaced with fresh complete culture medium.

#### Cell Counting Kit-8 assay

All specific manipulations were executed as delineated in prior studies.^59^ Because THBS1 expression was significantly higher in ectopic tissues than in eutopic tissues, we conducted knockdown experiments in cells from ectopic tissues and overexpression experiments in eutopic tissues.

#### Transwell assay

Transwell assay was performed as previously described.^59^ Each cell sample was tested in duplicate. Photographs were taken by microscopy (Olympus, Tokyo, Japan).

#### Flow cytometry analysis

Cells were stained using a Cell Cycle Staining Kit or Annexin V-APC/7-AAD Apoptosis Kit (MultiSciences, China), according to the manufacturer’s instructions, and analyzed via flow cytometry using a FACS system (Becton Dickinson, USA).

### Quantification and statistical analysis

The bioinformatic analysis was conducted in R programming version 4.3 (https://www.r-project.org/). Statistical analyses and graphing were performed using GraphPad Prism 8 and SPSS 17.0. Comparisons between two groups were performed using the Student’s *t* test. Comparisons between three groups were performed using the one-way ANOVA, in conjunction with Dunn’s post hoc test. Results are presented as mean ± standard deviation (Mean ± S.D.). Differences were considered significant at *p* < 0.05. Statistical significance was further denoted by asterisks: ∗*p* < 0.05,∗∗*p* < 0.01, ∗∗∗*p* < 0.001, ∗∗∗∗*p* < 0.0001.
