# Supplementary material for: THBS1 identificated as an endometriosis biomarker through evidence from single-cell and bulk transcriptomic profiling
Source: iScience. 2025 Nov 21;28(12):114164. doi: 10.1016/j.isci.2025.114164 (PMC12721194; doi:10.1016/j.isci.2025.114164)
Supplement: Document S1. Figures S1 [file mmc1.pdf]

iScience, Volume *28*

Supplemental information

THBS1 identificated as an endometriosis biomarker through evidence from single-cell and bulk transcriptomic profiling

Liqi Zhang, Junyan Ma, Yuhui Sun, Jue Zhu, Huaqing Yan, Yichen Chen, and Jing Zhang

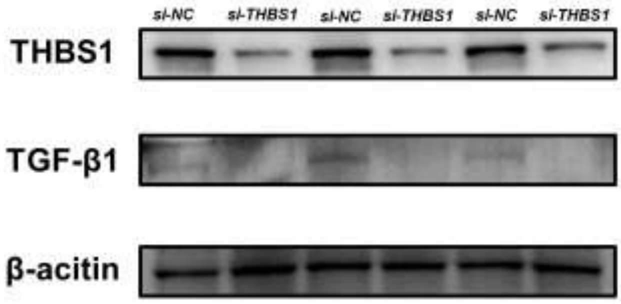

Supplementary Figure 1: Western blot analysis was performed in cells from ectopic endometrium tissues and demonstrated a marked reduction in TGF-β1 expression following si-THBS1 transfection.
